# Dilemmas in the Management of Osteoporosis in Younger Adults

**DOI:** 10.1002/jbm4.10594

**Published:** 2022-01-19

**Authors:** Madhuni Herath, Adi Cohen, Peter R. Ebeling, Frances Milat

**Affiliations:** ^1^ Department of Endocrinology Monash Health Clayton Victoria Australia; ^2^ Centre for Endocrinology & Metabolism Hudson Institute of Medical Research Clayton Victoria Australia; ^3^ Department of Medicine, School of Clinical Sciences Monash University Clayton Victoria Australia; ^4^ Department of Medicine Columbia University College of Physicians & Surgeons New York NY USA

**Keywords:** OSTEOPOROSIS, FRACTURE, BONE DENSITY, BONE QUALITY, PREMENOPAUSAL, YOUNG ADULT, FRACTURE RISK ASSESSMENT, FRACTURE PREVENTION, SCREENING, THERAPEUTICS

## Abstract

Osteoporosis in premenopausal women and men younger than 50 years is challenging to diagnose and treat. There are many barriers to optimal management of osteoporosis in younger adults, further enhanced by a limited research focus on this cohort. Herein we describe dilemmas commonly encountered in diagnosis, investigation, and management of osteoporosis in younger adults. We also provide a suggested framework, based on the limited available evidence and supported by clinical experience, for the diagnosis, assessment, and management of osteoporosis in this cohort. © 2021 The Authors. *JBMR Plus* published by Wiley Periodicals LLC on behalf of American Society for Bone and Mineral Research.

## Introduction

Osteoporosis has historically been a disease associated with postmenopausal women and elderly men. The high fracture incidence and associated mortality^(^ ^1^ ^)^ has led to a research focus on postmenopausal osteoporosis, with therapeutics tailored to this population. Fractures and osteoporosis in younger adults (YAs: premenopausal women and men aged <50 years) are less common but do occur in the setting of chronic disease, medications that affect bone metabolism, and other risk factors.^(^ ^2^ ^)^ Although rare, idiopathic osteoporosis (IOP) with no identifiable secondary cause is also associated with abnormal bone microarchitecture.^(^ ^3^ ^)^

Dilemma 1: The pathophysiology of fracture in YAs is poorly understood.

Dilemma 2: The BMD criteria for diagnosis of osteoporosis in YAs are debated. Osteoporosis in young adults can be diagnosed on the basis of low‐trauma fracture. In contrast, bone mineral density (BMD) criteria for diagnosis are debated. The 2019 International Society for Clinical Densitometry Position (ISCD) recommends against the use of dual‐energy X‐ray absorptiometry (DXA) alone in diagnosing osteoporosis in men and women <50 years of age,^(^ ^4^ ^)^ and states that the use of a *Z*‐score ≤ −2 on DXA can be used as a marker of “low BMD” in YAs. In contrast, the International Osteoporosis Foundation (IOF) recommends the use of *T*‐score ≤ −2.5 on DXA at the lumbar spine or hip to diagnose osteoporosis in YAs with a known secondary cause of osteoporosis,^(^ ^5^ ^)^ consistent with the World Health Organization criteria for osteoporosis in postmenopausal women and older men. The lack of united and consistent guidance on the diagnosis of osteoporosis in YAs adds a barrier to optimal osteoporosis care.

Dilemma 3: Optimal investigations for the diagnosis and monitoring of osteoporosis in YAs are unclear.

Dilemma 4: Best practice management of osteoporosis or low bone density in YAs is poorly understood due to scarcity of focused research. This is also reflected in the lack of guidelines that address risk stratification for fracture and osteoporosis in YAs.

## Objective

We provide a systematic overview of the current understanding of the physiology of bone accrual and factors affecting peak bone mass, the potential secondary causes of osteoporosis that may disrupt the attainment of optimal peak bone mass and also accelerate bone loss, and the entity of IOP. We also discuss the current dilemmas in the diagnosis, risk stratification, and management of osteoporosis in YAs.

## Methods

A literature search for articles published in the English Language between January 2000 and August 2021 was conducted using predetermined keywords (see Appendix S1) on PubMed, Embase, and Web of Science. This timeframe was chosen to identify the most relevant research findings published in recent times. A manual search of cited references in relevant articles was also conducted to identify further studies. Following full‐text review, relevant and eligible articles were included in this narrative review; systematic reviews that included studies outside of the selected time range were not excluded.

### Physiology of bone accrual and bone mass trajectory in healthy adolescents and adults

**Fig. 1:**
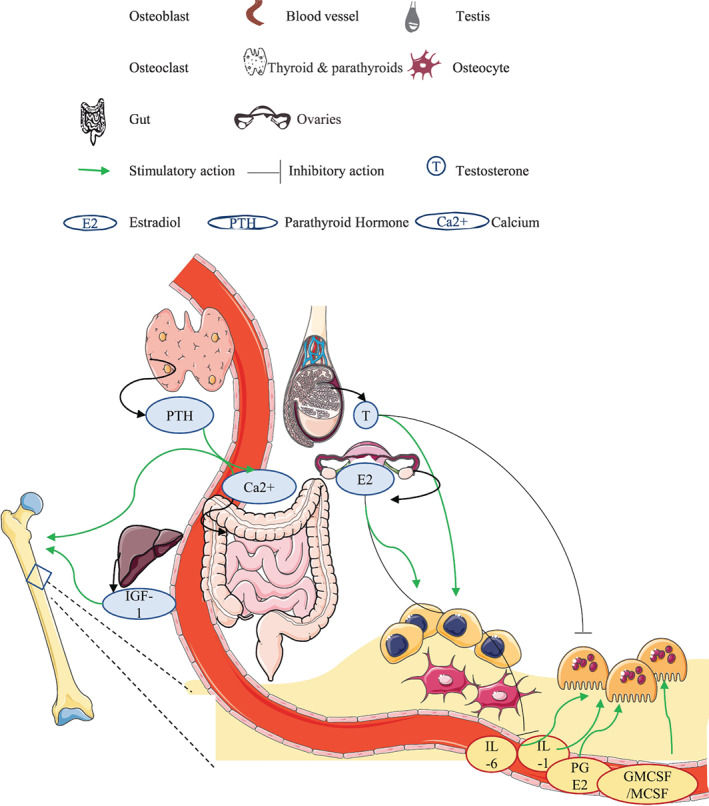
Physiological bone accrual in young adulthood. Estrogen inhibits pro‐inflammatory cytokines and osteoclast action while promoting osteoblast function. Testosterone promotes osteoblast function by inhibiting apoptosis and inhibits osteoclast function. IGF‐1, parathyroid hormone also promote bone accrual, in addition to other hormonal and mechanical influences which regulate the attainment of bone mass in young adult years.

Sex steroids play a major role in bone accrual across the lifespan. Estrogen decreases osteoclast activity and increases osteoblast activation (Fig. 1).^(^ ^6^ ^)^ This action is partly actioned by osteoprotegerin (OPG), which is increased in the presence of estrogen and inhibits receptor activator nuclear factor κB ligand (RANKL), which would otherwise activate osteoclasts. Estrogen also has effects on the inflammatory cytokines interleukin‐1 (IL‐1), IL‐6,^(^ ^7^ ^)^ granulocyte colony‐stimulating factor (G‐CSF), macrophage colony stimulating factor (M‐CSF), and prostaglandin E2 (PG‐E2), all of which act to increase bone resorption.^(^ ^6^ ^)^ Testosterone inhibits osteoblast apoptosis, increases proliferation, and promotes apoptosis of osteoclasts.

Bone growth accelerates during puberty. High‐resolution peripheral quantitative computed tomography (HRpQCT) studies demonstrate that postpubertal girls have higher cortical density at the radius, whereas postpubertal boys have higher trabecular volume and larger cortical cross‐sectional areas than girls. Postpuberty, mature girls and boys have increased cortical BMD and less cortical porosity than their less mature counterparts within each sex.^(^ ^8^ ^)^ The increased trabecular bone parameters are a phenomenon thought to be driven by both testosterone and insulin‐like growth factor‐1 (IGF‐1). This supports the observation that men achieve higher peak bone mass secondary to the development of larger bones through greater periosteal apposition.

Genetic, biological, environmental, and lifestyle factors all modify peak bone mass in YAs. Low body weight,^(^ ^9^, ^10^ ^)^ delayed puberty,^(^ ^9^, ^11^ ^)^ and lack of weight‐bearing physical activity^(^ ^9^, ^10^ ^)^ are associated with lower areal (aBMD) or volumetric BMD (vBMD). Physically active male adolescents develop greater total area at the distal tibia and adjusted torsional tibial strength, and physically active females develop greater cortical area and density as well as trabecular content at the tibia compared to inactive adolescents.^(^ ^10^ ^)^ Tobacco smoking is also associated with reduced spinal and hip aBMD as well as reduced tibial trabecular vBMD.^(^ ^12^ ^)^

Normal pregnancy and lactation are associated with rapid asymptomatic decreases in BMD followed by recovery during and after weaning. Interpretation of BMD results obtained after pregnancy in premenopausal women should take into account these normal and expected changes. Some studies suggest that reproductive changes may ultimately have lasting structural benefits. In a cohort of young women,^(^ ^9^ ^)^ a history of pregnancy was associated with higher spine aBMD and vBMD. Pregnancy and breastfeeding history also do not appear to increase risk of postmenopausal osteoporosis. In an analysis of data from >92,000 women included in the Women's Health Initiative Observational Study and followed for a mean of 7.9 years, incident hip fracture was not associated with number of pregnancies, or duration of breastfeeding in adjusted analyses,^(^ ^13^ ^)^ and breastfeeding history was associated with 16% lower risk of hip fracture.

Declines in bone mass, especially trabecular vBMD loss, commences from the third decade of life in both sexes, and are accelerated postmenopause in women.^(^ ^14^ ^)^ Cortical bone loss commences from the fifth to sixth decade of life. Postmenopausal women experience increased bone resorption and trabecular loss, whereas men experience trabecular thinning due to reduced bone formation, with age. By late menopause cortical bone density, absolute cortical zone thickness, trabecular bone density, and the relative bone volume as part of total volume are all reduced, even in healthy women.^(^ ^15^ ^)^

### Dilemma 1: Gaps in understanding the pathophysiology of osteoporosis in YAs

#### Secondary osteoporosis in YAs

Chronic disease during childhood and young adulthood can interfere with accrual of peak bone mass through various mechanisms. Increased exposure to inflammatory cytokines, poor nutrition, weight loss, hypogonadism, and other disease‐specific mechanisms promote bone loss. The most common etiologies for bone loss in YAs are listed in Table 1 and are discussed below.

**Table 1:** Conditions that Increase Risk of Osteoporosis in Younger Adults

Inflammatory/autoimmune disease Systemic lupus erythematosus Rheumatoid arthritis Cystic fibrosis Crohn's disease Ulcerative colitis Ankylosing spondylitis Endocrine dysfunction Cushing's syndrome (iatrogenic or due to organic pathology) Hypogonadism and functional hypothalamic amenorrhea Type 1 diabetes mellitus Growth hormone deficiency Hyperthyroidism Hyperparathyroidism Complete androgen insensitivity Subtherapeutic transgender hormone therapy Malabsorptive disease Celiac disease Psychiatric disease Schizophrenia Anorexia nervosa Other Cancer and cancer‐treatment Gastric bypass surgery Thalassemia Human immunodeficiency virus Systemic mastocytosis Solid organ or bone marrow stem cell transplant

##### Glucocorticoid‐induced osteoporosis

Glucocorticoid‐induced osteoporosis (GIO) can occur in the context of excess glucocorticoids due to Cushing syndrome^(^ ^16^ ^)^ and in those prescribed long‐term, moderate‐dose to high‐dose glucocorticoids (equivalent to ≥5 mg prednisolone for ≥3 months). Treatment of the underlying disease is recommended in those with endogenous Cushing syndrome.

Glucocorticoids induce increased bone resorption and reduce bone formation^(^ ^17^ ^)^ through impairment of osteoblast maturation and osteoclast apoptosis.^(^ ^18^ ^)^ Many interventional studies have been performed to identify suitable therapy for GIO, although almost none examined the effects of these agents exclusively in a younger adult cohort. Zoledronic acid (ZA) has proven efficacy in treating GIO.^(^ ^19^ ^)^ Denosumab^(^ ^20^, ^21^ ^)^ increases spinal and femoral neck BMD and is superior to risedronate. Teriparatide is superior to risedronate^(^ ^22^ ^)^ and alendronate,^(^ ^23^ ^)^ and improves fracture risk in addition to benefits in aBMD and vBMD. Teriparatide remained superior when outcomes were assessed in postmenopausal and premenopausal women separately.^(^ ^24^ ^)^ In premenopausal patients with GIO and systemic lupus erythematosus (SLE), alendronate and calcium increased spine and hip BMD significantly more than calcium alone, or concurrent calcium and calcitriol supplementation.^(^ ^25^ ^)^ Intravenous pamidronate improved spinal and hip BMD over 12 months in 16 premenopausal women with connective tissue disease and GIO.^(^ ^26^ ^)^ A systematic review and network meta‐analysis of 27 randomized controlled trials (RCTs) demonstrated that teriparatide, risedronate, and etidronate were effective in preventing vertebral fracture in patients with GIO, compared with calcium and vitamin D; fracture data for denosumab were unavailable. Teriparatide, zoledronic acid, risedronate, alendronate, and etidronate improved spinal BMD in GIO compared with calcium and vitamin D, whereas calcitonin did not; alendronate and raloxifene improved femoral neck BMD.^(^ ^27^ ^)^ A more recent systematic review of denosumab treatment in GIO demonstrated an increase in spinal (4.59%) and total hip (2.54%) BMD at 12 months.^(^ ^28^ ^)^ When compared to bisphosphonates, denosumab increased spinal BMD by 2.17% at 12 months. Denosumab was more efficacious than bisphosphonates at increasing femoral neck BMD at 12 months (0.97%); fracture data was not available. The study by Lambrinoudaki and colleagues,^(^ ^29^ ^)^ investigating the efficacy of calcitriol and calcium supplementation in SLE, was the only study included in the meta‐analysis which exclusively evaluated YAs, and demonstrated no benefit. Five others assessed populations with a mean age <50 years. These demonstrated that risedronate protected against spinal aBMD loss in GIO in rheumatologic disease,^(^ ^30^, ^31^ ^)^ and was superior to calcium and vitamin D supplementation alone in Crohn's disease.^(^ ^32^ ^)^ Calcium and vitamin D supplementation alone was not beneficial at 1 year in patients with Crohn's disease^(^ ^33^ ^)^ and neither was ibandronate in comparison with placebo, in SLE.^(^ ^34^ ^)^

##### Endocrine dysfunction

Hypogonadism is an established risk factor for bone loss. Premature ovarian insufficiency, primary testicular failure due to conditions including Klinefelter syndrome, and hypogonadotropic hypogonadism cause bone loss. Impact on BMD and fracture risk is particularly increased in men and women who are diagnosed late, or where there is a delay in commencing hormone replacement therapy.^(^ ^35^ ^)^ Although driven by hypogonadism, bone loss in these individuals is multifactorial due to the impact of these diseases on body composition and other associated comorbidities. The effects of these pathologies on bone health are outside the scope of this review. The musculoskeletal effects of premature ovarian insufficiency have been reviewed elsewhere.^(^ ^36^, ^37^ ^)^

Similarly, hypogonadism in the setting of cancer treatment is a risk factor for fragility fracture.^(^ ^38^, ^39^ ^)^ Use of gonadotropin‐releasing hormone agonists, aromatase inhibitors, chemotherapeutic agents such as cyclophosphamide, gonadectomy, and pelvic or cranial radiotherapy increases risk of bone loss. Evidence for prevention of bone loss with intervention in YAs are limited. In premenopausal women with breast cancer, combined aerobic and circuit training can prevent bone loss at the femoral neck.^(^ ^40^ ^)^ Six‐monthly ZA also prevents bone loss associated with concurrent aromatase inhibition and ovarian suppression in premenopausal women,^(^ ^41^ ^)^ whereas 3‐monthly administration prevents bone loss secondary to chemotherapy‐induced ovarian failure.^(^ ^42^ ^)^ Weight‐bearing exercise, and optimization of calcium and vitamin D is currently recommended in women receiving endocrine therapy for estrogen‐receptor positive breast cancer.^(^ ^43^ ^)^ ZA is recommended if *Z*‐score is ≤ −2 at any site or if annual bone loss is >5%. ZA also preserved BMD in a small group of adults including men with testicular cancer and lymphoma. However, like most studies, this included men from a broad age range (21–78 years).^(^ ^44^ ^)^

Functional hypothalamic amenorrhea is another complex cause of osteoporosis in young, female adults and can affect elite athletes. Suppression of the hypothalamic‐pituitary‐gonadal axis is responsible for the disruption to menstruation, estrogen balance, and bone accrual and turnover. At non‐weight‐bearing sites, amenorrheic adolescent and YA females have lower stiffness and failure load than eumenorrheic controls.^(^ ^45^ ^)^ Transdermal estrogen replacement following adequate nutrition and weight restoration to healthy, pre‐amenorrheic levels benefit these women and are recommended.^(^ ^46^ ^)^ However, the use of the oral contraceptive pill does not appear to benefit bone health.^(^ ^46^, ^47^ ^)^

Type 1 diabetes mellitus (T1DM) results in increased fracture risk in YAs.^(^ ^48^ ^)^ In a systematic review and meta‐analysis, relative risk of hip fracture was 4.4 in young and middle‐aged adults with T1DM. Reduced aBMD is not always present, despite increased fragility fracture.^(^ ^49^ ^)^ Young women with T1DM also have reduced tibial trabecular density and cortical thickness.^(^ ^50^ ^)^ In YAs, long‐term glycated hemoglobin (A1c) levels correlate with fracture prevalence.^(^ ^49^ ^)^ However, other than case reports, there are currently no studies that examine the effect of anti‐resorptive therapy in YAs with T1DM.

Hyperthyroidism is also associated with reduced BMD in young premenopausal women,^(^ ^51^ ^)^ and correction of hyperthyroidism is recommended. Childhood‐onset growth hormone deficiency untreated since completion of skeletal growth is associated with reduced tibial cortical and trabecular vBMD as well as increased trabecular separation.^(^ ^52^ ^)^

Complete androgen insensitivity syndrome (CAIS), most commonly 46XY DSD, is caused by mutations in the androgen receptor, resulting in complete androgen resistance in androgen‐dependent tissues. Despite hormone replacement therapy for a mean of 17.4 years, reduced spinal, femoral neck, and total body BMD is noted in women with CAIS who have undergone gonadectomy during their early adolescence.^(^ ^53^ ^)^ Oral and transdermal estrogen replacement therapy (2 mg transdermal estradiol or 2 mg oral estradiol valerate) was associated with a small but significant (4.2%) improvement in spinal aBMD over 6 years, in a small subgroup of women in this cohort. Femoral neck and total body BMD were unaffected.

The field of bone health in transgender men and women is gathering momentum. Transwomen appear to gain aBMD with hormonal therapy, whereas reports of both bone loss and stability have been reported in transmen.^(^ ^54^, ^55^ ^)^ A systematic review and meta‐analysis found no significant change in aBMD in transmen following hormonal therapy^(^ ^56^ ^)^; however, the quality of evidence was low. Screening for osteoporosis is recommended in those who cease therapy or are noncompliant.^(^ ^57^ ^)^ For transwomen considered low risk (ie, no prior fragility fracture or other clinical risk factors for osteoporosis, adherent to gender‐affirming hormone therapy regardless of gonadectomy status), screening is recommended at 60 years of age; consideration of DXA at commencement of hormonal therapy is recommended for others.^(^ ^57^ ^)^

##### Anorexia nervosa

YAs with anorexia nervosa (AN) have multiple risk factors for bone loss, including low body weight, poor nutrition, hypogonadism, and other hormonal abnormalities. Compared with healthy controls, young women with AN have considerably lower number of trabeculae and higher trabecular separation, similar to the findings observed in postmenopausal women.^(^ ^15^ ^)^ In adolescents and young women, AN is associated with resistance to axial and bending loads on hip structural analysis (HSA).^(^ ^58^ ^)^ A study of men with eating disorders, aged 18 to 65 years confirmed low spinal and hip BMD in men with AN.^(^ ^59^ ^)^ Disease duration, lower body mass index (BMI), muscle mass, and vitamin D levels were associated with low BMD.^(^ ^59^ ^)^ Reduced trabecular bone score (TBS), identified in 13% of women in a subgroup with AN and lower BMI than the rest of the cohort, suggests worsened bone quality in those with lower weight.^(^ ^60^ ^)^ Improvement of BMD in these women are challenging, and rely on weight restoration^(^ ^61^, ^62^, ^63^ ^)^ and resumption of menses.^(^ ^62^, ^63^ ^)^ A cross‐sectional study of women revealed that moderate‐intensity exercise while unwell with active AN worsens BMD. In contrast, in patients who have recovered from AN, lifetime moderate‐intensity exercise levels had a positive effect on BMD.^(^ ^64^ ^)^ The oral contraceptive pill was found not to be beneficial in restoring bone in young women.^(^ ^63^ ^)^ Although the effects of transdermal estradiol have not been consistent,^(^ ^62^ ^)^ there is evidence for aBMD improvements with doses of 0.045 to 0.1 mg 17B estradiol in addition to cyclical progesterone in girls^(^ ^65^ ^)^ and young women.^(^ ^66^ ^)^ Furthermore, an RCT of risedronate, transdermal testosterone at 150 μg daily, combination therapy, or double placebo in 77 young women with AN demonstrated improvements in spine (3%) and hip (2%) BMD over 12 months in those treated with risedronate.^(^ ^67^ ^)^ Transdermal testosterone did not have a detectable response. Teriparatide increased spinal aBMD at 6 months in women (mean ± standard deviation [SD] age 47 ± 2.7 years) with AN.^(^ ^68^ ^)^ Benefits for testosterone, anabolic, and anti‐resorptive therapy in men with AN are not well‐documented.

##### Autoimmune or inflammatory disease

YAs with inflammatory disease are at risk of osteoporosis and fracture due to changes caused by the underlying disease and increased exposure to inflammatory cytokines and medications that promote bone loss.

In premenopausal women with SLE, osteopenia or osteoporosis is highly prevalent.^(^ ^69^ ^)^ Premenopausal women experience greater loss of hip aBMD as well as cortical area and thickness over 24 months, compared with healthy premenopausal women. Increased cortical porosity is also noted.^(^ ^70^ ^)^ SLE has been shown to contribute to bone loss, even in the absence of glucocorticoid exposure.^(^ ^71^ ^)^ This study, however, included adults aged ≥50 years (mean 46 years). In a subgroup analysis of premenopausal women, significant effects of SLE on cortical bone was identified. Total hip aBMD and radial cortical area was reduced in premenopausal women unexposed to glucocorticoids, while cortical area and thickness was reduced in those exposed. The utility of anti‐resorptive and anabolic therapy in treating osteoporosis in SLE are derived from studies investigating GIO and were discussed under section heading 'Glucocorticoid‐induced osteoporosis'.

Inflammatory bowel disease (IBD) affects YAs, with a significant impact on bone health. GIO,^(^ ^72^ ^)^ malabsorption in the setting of gut inflammation and bowel resection,^(^ ^72^ ^)^ and hypogonadism^(^ ^73^ ^)^ are additional risk factors for bone loss. Many studies include YAs, but do not exclusively study them. In a cohort of adults with a mean age of 40.6 years, osteopenia and osteoporosis was prevalent in >50% of individuals with IBD.^(^ ^72^ ^)^ In comparison with a healthy population, a >40% increased prevalence of vertebral and hip fractures was observed, even following subgroup analysis of YA cohorts.^(^ ^74^ ^)^ Minimization of glucocorticoids and preferential use of tumor necrosis factor α (TNF‐α) blockers^(^ ^75^ ^)^ may help preserve bone; however, studies that evaluate the effect of these interventions in YAs are required.

YAs with cystic fibrosis (CF) have higher risk of fracture, secondary to malabsorption of calcium and vitamin D,^(^ ^76^ ^)^ lower body weight,^(^ ^77^ ^)^ delayed puberty,^(^ ^77^ ^)^ GIO,^(^ ^77^ ^)^ poor nutrition,^(^ ^78^ ^)^ limited physical function,^(^ ^79^ ^)^ chronic inflammation, and underlying impact of the CF transmembrane conductance regular (CFTR) gene mutation.^(^ ^80^ ^)^ Up to 52% of YAs with CF may have associated bone disease,^(^ ^81^, ^82^ ^)^ with a higher prevalence noted in cohorts of patients with end‐stage lung disease.^(^ ^76^ ^)^ The severity of disease as measured by forced expiratory volume in 1 second (FEV1) on lung function testing, also correlates with spinal aBMD.^(^ ^79^, ^83^ ^)^ Markers of bone formation correlate negatively with IL‐6 and C‐reactive protein (CRP), highlighting the impact of inflammation. HRpQCT studies demonstrate reduced trabecular vBMD, thickness, and number, and greater trabecular separation.^(^ ^84^ ^)^ Current guidelines recommend DXA in all adults and children aged >8 years of age if FEV1 is <50% predicted, glucocorticoid use is equivalent to 5 mg daily prednisolone for ≥90 days/year, or in the event of pubertal delay or history of fracture.^(^ ^85^ ^)^ For *T*‐scores or *Z*‐scores ≤ −2, consideration of bisphosphonates is recommended after nutritional deficiencies and pulmonary and endocrine pathology have been optimized. Fractures may lead to consideration of anabolic osteoporosis therapy in YAs. Utility of targeted CFTR therapy on bone parameters are underway. Ivacaftor treatment is associated with increases in cortical bone inpatients aged 19 to 75 years with G551D mutations of the CFTR gene.^(^ ^86^ ^)^

Systemic mastocytosis is a rare cause of osteoporosis in YAs. However, case series of fractures in YAs with systemic mastocytosis do exist, and consideration as a secondary cause of osteoporosis is recommended.^(^ ^87^ ^)^

##### Hematologic disease

There is irrefutable evidence that transfusion‐dependent thalassemia major results in osteoporosis and increased fracture risk. Risk factors for low BMD in this group include hypogonadism, marrow expansion, iron toxicity and chelators, and increased bone turnover. The contribution of renal dysfunction to bone disease in thalassemia is also evolving, with renal tubular dysfunction and hypercalciuria associated with the chelating agent deferasirox.^(^ ^88^ ^)^ Individuals should also be screened and treated for vitamin D deficiency. Optimizing red blood cell transfusion and the addition of bisphosphonates (once osteomalacia is excluded) assist with bone preservation.^(^ ^89^ ^)^ Correction of hypogonadism is important and supported by the available literature, but the optimal dose and route for hormone therapy is unclear.

##### Malabsorptive disorders

Untreated celiac disease is associated with increased fracture risk by up to 43% and abnormal bone microarchitecture.^(^ ^90^ ^)^ Malabsorption secondary to celiac disease also causes vitamin D deficiency. Although a gluten‐free diet, and vitamin D and calcium supplementation^(^ ^91^ ^)^ improve bone microarchitecture, complete restoration was not observed.

##### Human immunodeficiency virus

A systematic review and meta‐analysis has revealed a 50% higher fracture risk in adults with human immunodeficiency virus (HIV).^(^ ^92^ ^)^ Prior fracture, postmenopausal status, co‐infection with hepatitis C virus, and the presence of an acquired immunodeficiency syndrome (AIDS)‐defining illness was associated with an increased fracture risk.^(^ ^93^ ^)^ Antiretroviral therapy, particularly containing tenofovir disoproxil fumarate, is also associated with increased risk of bone loss, although studies included adults aged >50 years.^(^ ^94^, ^95^ ^)^

##### Gastric bypass surgery

Both Roux‐en‐Y gastric bypass (RYGB) surgery and sleeve gastrectomy contribute to bone loss. In an observational study of premenopausal women who underwent RYGB or sleeve gastrectomy, spinal and hip aBMD loss was significant and persistent (~18% total loss in BMD).^(^ ^96^ ^)^ Increased fracture rates are also reported, up to 15 years post–bariatric surgery in a group of adults aged 44 ± 10 years at the time of surgery.^(^ ^97^ ^)^ Because the bone loss persists at 6 months despite weight stabilization, it is hypothesized that hormonal influences contribute to ongoing deterioration in bone.^(^ ^98^ ^)^

##### Solid organ and bone marrow stem cell transplantation

Studies which exclusively investigate YAs are limited in this population. In stem cell transplantation (SCT) recipients for hematological malignancies, early loss of BMD at the femoral neck compared with the lumbar spine^(^ ^99^ ^)^ persists for 12^(^ ^100^ ^)^ to 36^(^ ^101^ ^)^ months. This is further highlighted in a study of 47 adults with a mean age of 43 years at the time of bone marrow transplant (73% of women were premenopausal), because the development of osteopenia or osteoporosis at the hip was identified in up to 47% of patients by 12 to 14 months following transplantation.^(^ ^100^ ^)^ Glucocorticoid therapy, exposure to chemotherapeutic agents such as cyclosporine A,^(^ ^99^ ^)^ and chronic graft versus host disease is associated with bone loss. Three doses of 3‐monthly intravenous ZA infusions improve spinal and femoral aBMD (9.8% and 6.47%, respectively), 12 months following first infusion^(^ ^101^ ^)^; oral risedronate also protects spine BMD compared with controls.

Solid‐organ transplant is associated with increased fracture and BMD loss especially in the first 6 months following transplantation; bone recovery is observed in some patient post–liver transplant over a longer‐period of time.^(^ ^102^ ^)^ Both alendronate and calcitriol prevented femoral aBMD loss in adults (aged 18–71 years) post–cardiac transplant in comparison to an untreated reference group; no significant difference was identified between the calcitriol‐treated and alendronate‐treated groups.^(^ ^103^ ^)^ In adults aged 18 to 70 years, one infusion of 5 mg ZA and weekly alendronate were both efficacious in preserving hip aBMD and increasing spinal aBMD in liver transplant patients, while in cardiac transplant patients given alendronate, spinal aBMD decreased.^(^ ^104^ ^)^ ZA protected spinal aBMD in patients with cardiac transplant. Alendronate failed to protect spinal aBMD.^(^ ^104^ ^)^

##### Psychiatric disorders

Studies in YAs with schizophrenia have revealed lower aBMD compared with healthy controls. The impact of secondary risk factors separate from the underlying disease on bone health has not been delineated. Some studies do demonstrate reduced BMD^(^ ^105^ ^)^ in YAs presenting with their first episode of psychosis, while others do not.^(^ ^106^ ^)^ In a cross‐sectional study of YAs with schizophrenia receiving long‐acting risperidone, reduced BMD was not noted, although a slight but significant increase in carboxy‐terminal cross‐linking telopeptide of type 1 collagen (CTx) levels was observed.^(^ ^107^ ^)^

Adults prescribed antidepressants such as selective serotonin reuptake inhibitors (SSRIs) are also at high risk of fracture.^(^ ^108^ ^)^ YAs with psychiatric disease continue to be an underserved cohort with multiple risk factors for falls and fracture; their bone disease and endocrine complications require further elucidation.

#### IOP

Some patients present with early‐onset osteoporosis in the absence of an identifiable cause. Clinical presentations vary, and affected men and women may have many different underlying etiologies rather than a unifying single cause. Young adults presenting with early‐onset osteoporosis in the absence of a secondary cause may also have a primary or genetic etiology of bone fragility.

##### Pathophysiology

In premenopausal women with IOP, bone remodeling rate is variable. The lack of consistently low or high bone turnover in IOP suggests the underlying etiologies are heterogeneous. Osteoblast dysfunction is a potential cause of IOP in YAs. A subgroup with the lowest bone formation were found to have the most profound microarchitectural deficiencies.^(^ ^3^ ^)^ Lower trabecular bone mineralization with elevated proteoglycan content and collagen crosslink ratio quantified through Fourier transform infrared microspectroscopy of transiliac biopsies also support the hypothesis of osteoblast dysfunction.^(^ ^109^ ^)^ In HRpQCT studies, women with IOP had lower trabecular vBMD and tibial cortical density and thickness.^(^ ^110^ ^)^ The relationship between “plate”‐like and “rod”‐like structures within the individual trabeculae, which correlate with bone strength, is also impaired. In IOP, there are reduced trabecular plates and rods as well as connections between these structures, potentially leading to a less dense, less connected structure, leading to reduced bone strength.^(^ ^111^ ^)^ Importantly, although women with IOP and fracture had lower aBMD than controls, the mean *Z*‐score for spinal aBMD was > −2,^(^ ^3^ ^)^ suggesting that mechanisms other than low aBMD may be contributing to the bone fragility or poor bone quality.

In men, transiliac biopsies demonstrate reduced cortical and trabecular bone volume with no change in cortical porosity, consistent with reduced osteoblast availability.^(^ ^112^ ^)^ Sclerostin, an inhibitor osteoblast differentiation and the Wnt signaling pathway for bone formation does not appear to be responsible because levels were lower in men with IOP.^(^ ^113^ ^)^ Low IGF‐1 levels have, however, been reported, and may have a role in IOP in men.^(^ ^114^ ^)^

Increased bone marrow adiposity^(^ ^115^ ^)^ has been described in premenopausal women with IOP. Their implications for bone health in this cohort require further evaluation.

A genetic basis for the pathophysiology of IOP has been suggested. Reported family history of osteoporosis is common in premenopausal IOP.^(^ ^116^ ^)^ Twin studies and observations that sons and brothers of men with IOP aged ≤65 years have a high prevalence of low spinal aBMD^(^ ^117^ ^)^ support this hypothesis. Single nucleotide polymorphisms at the osteonectin gene^(^ ^118^ ^)^ and mutations at a few other genetic loci^(^ ^119^ ^)^ including the *LRP5* gene have been suggested as potential culprits.^(^ ^120^ ^)^ However, the mechanisms through which these variants affect bone are is yet unclear—whether it is through an effect on bone accrual, peak bone mass, or early age‐related bone loss. Interestingly, adults with *LRP5* variants have reduced tibial and radial trabecular number and lower hip aBMD than at the spine, whereas those with *LRP6* variants have lower spinal aBMD.^(^ ^121^ ^)^

#### Monogenetic etiologies of early‐onset osteoporosis

Monogenetic or primary osteoporosis classically presents in childhood. However, clinical severity of some genetic forms of osteoporosis can be variable, and patients can have an initial presentation in young adulthood. Presentation with multiple fragility fractures in YA may lead to consideration and evaluation for primary osteoporosis or monogenetic disorders, such as osteogenesis imperfecta, hypophosphatasia syndromes, X‐linked osteoporosis, and osteoporosis‐pseudoglioma syndrome. Although rare, there is clearly defined increased risk of fracture in these syndromes.

Some patients with IOP may have an as yet undiagnosed primary or monogenetic cause of osteoporosis. In a study of 123 men and women with IOP, candidate gene sequencing identified 11 patients with rare or novel variants in *COL1A2*, *PLS3*, *WNT1*, or *DKK1*, plus an additional 16 patients with very rare or novel variants in *LRP5*.^(^ ^120^ ^)^ In a cohort of 75 women with IOP or idiopathic low bone mass, whole‐exome sequencing identified eight subjects with heterozygous likely pathogenic variants or variants of undetermined significance in relevant genes (*LRP5*, *PLS3*, *FKBP10*, *SLC34A3*, and *HGD*).^(^ ^122^ ^)^ Some variants identified pointed to defects in bone formation, whereas others pointed to high bone turnover etiologies. Of note, the great majority of subjects had no identifiable genetic etiology. Future research may identify new genetic causes of IOP.

#### Pregnancy and lactation associated osteoporosis

Pregnancy and lactation are associated with physiologic changes in bone mass and increased calcium demand. Rarely, fragility fractures occur in the context of these normal and expected changes. This condition, pregnancy and lactation associated osteoporosis (PLO), is characterized by spontaneous or fragility fractures, commonly affecting the spine and other trabecular sites, during late pregnancy or lactation. Etiology and predisposing factors require elucidation and are under investigation. Most reported cases have no known secondary cause of osteoporosis. In a recently described cohort of >100 women with PLO, 70% were primiparous, 95% were diagnosed during lactation, and >85% presented with vertebral fractures (mean number of fractures 4 ± 2).^(^ ^123^ ^)^ Longitudinal follow‐up over a median of 6 years suggested ongoing high fracture risk. Case reports have described benefits of bisphosphonates for women with PLO. In an observational study, 12 months of teriparatide led to increases (15.5 ± 6.6%) in spinal aBMD in 27 women treated, significantly more than increases seen in five untreated controls (7.5 ± 7.1).^(^ ^124^ ^)^ Other, smaller studies of teriparatide have demonstrated similar results at the spine and hip, following 12 to 24 months of treatment.^(^ ^125^ ^)^

### Dilemma 2: The BMD criteria for the diagnosis of osteoporosis in YAs is unclear

The ISCD and IOF recommend diagnosis of low BMD or osteoporosis based on *Z*‐scores or *T*‐scores, respectively, in YAs with underlying secondary causes.^(^ ^4^, ^5^ ^)^ Others have recommended against diagnosing osteoporosis in the absence of fragility fractures.^(^ ^85^ ^)^ However, the latter raises challenges of its own, because treatment following fracture, rather than fracture primary prevention, is prioritized. To add complexity, studies reporting the magnitude of fracture risk in isolated low BMD measured by a *Z*‐score ≤ −2 are very limited.

### Dilemma 3: Optimal investigations for the diagnosis and monitoring of osteoporosis in YAs are unclear

Geographical and ethnic variation in aBMD in YAs requires due consideration. Singaporean Chinese premenopausal women aged 40 to 50 years have higher spinal aBMD compared with women of similar age from white, Japanese, Korean, and Thai populations.^(^ ^126^ ^)^ Radial cortical thickness and trabecular bone indices such as thickness, bone volume fraction, and plate bone volume are lower in white premenopausal women compared to Chinese‐American women.^(^ ^127^ ^)^ It is known that the absolute fracture risk at any given BMD differs between ethnic groups in postmenopausal women; it is less clear, however, whether this discrepancy also affects YAs.^(^ ^128^ ^)^

It is also important to appreciate that adults with chronic disease may sustain fragility fractures in the absence of low aBMD on DXA. This is especially true in chronic disease where up to 30% of individuals with fracture have normal aBMD.^(^ ^129^, ^130^, ^131^, ^132^ ^)^ This is a significant limitation of current fracture risk assessment tools as used by health care providers.

There are also implications of diagnosing and managing osteoporosis using DXA in populations with skeletal variations that may impact DXA outcome. Short stature occurs commonly in CF and other childhood illnesses that affect growth and can lead to underestimated bone density on DXA, because it measures aBMD, not vBMD, and is therefore unable to adjust for height‐related changes. Rheumatological diseases such as ankylosing spondylitis affect the lumbar skeleton and can result in inaccurate outputs from DXA scans. This is especially true in advanced disease, when fracture risk may be even higher.

Advanced analyses such as TBS and HSA have demonstrated utility in researching the impact of chronic disease on bone quality and structure. However, there is insufficient evidence to recommend incorporation into routine clinical practice.

The utility of alternate modalities to DXA, such as HRpQCT and calcaneal ultrasound has also been assessed in smaller cohorts of younger adults. Evaluation of HRpQCT in 54 YAs demonstrated strong correlations between trabecular parameters of bone volume fraction, number, and separation, as well as tibial cortical thickness and micro‐finite element analysis with that of transiliac biopsy findings.^(^ ^133^ ^)^ Although emerging research continues to demonstrate the utility of HRpQCT in understanding microarchitecture in bone, it remains a research tool and is not widely available for clinical use. Calcaneal ultrasound is presented as a potential screening tool for osteoporosis, particularly in settings where accessibility to routine imaging techniques is limited. Cortical speed of sound levels on calcaneal ultrasound correlates with cortical tissue mineral density on HRpQCT.^(^ ^134^ ^)^ The correlation with spinal or hip aBMD is, however, limited. Although calcaneal ultrasound may provide information specific to cortical bone abnormalities, it is important to appreciate that these parameters have not shown optimal correlation with transiliac biopsy findings, and therefore its clinical utility remains limited.

Although some of these challenges with use of DXA are also acknowledged in older adults, it is important to appreciate that the underlying etiologies for osteoporosis in YAs are heterogeneous, resulting in the competing effects on reduced peak bone mass, ongoing bone accrual, and increased bone resorption. As a result, DXA may be a poorer predictor of fracture risk in YAs. There is also lack of prospective data delineating the relationship between BMD and fracture risk in YAs. Investigation of the utility of supplementary DXA analyses such as HSA and TBS as well as novel imaging modalities such as HRpQCT will inform whether this dilemma can be addressed through newer imaging methods.

The Fracture Risk Assessment Tool (FRAX®) and the Garvan fracture risk calculators cannot be used to assess fracture risk in YAs. FRAX^(^ ^135^ ^)^ has been validated for use in adults ≥40 years of age, and the Garvan fracture risk calculator^(^ ^136^ ^)^ in adults ≥50 years of age. Limited studies have demonstrated the applicability of FRAX in subpopulations of younger adults with chronic disease; however, a clear gap in available risk assessment tools for YAs remains.

### Dilemma 4: The optimal management of osteoporosis in YAs is unclear

Current management of osteoporosis in YAs relies heavily on studies that include postmenopausal women and older men. As demonstrated in Table 2, very few studies investigate the efficacy and safety of available therapies specifically in YAs. Most available studies use surrogate markers for fracture, such as aBMD or HRpQCT parameters.

**Table 2:** BMD and Fracture Outcomes in Studies Investigating Anti‐Osteoporosis Therapy Exclusively in Premenopausal Women and/or Men Aged <50 Years: Studies of Subjects with IOP and Some Primary and Secondary Etiologies

Authors	Year	Title	Population	Study design	Intervention and comparator	Follow‐up	Outcome	
							BMD outcomes	Fracture outcomes
AN								
Miller and colleagues^137^	2004	Effects of risedronate on bone density in anorexia nervosa	10 women with AN, compared to published data on 14 controls	Pre‐test post‐test clinical trial	Risedronate 5 mg daily	9 months	+4.1 ± 1.6% change in spinal BMD in women receiving risedronate versus −1.5 ± 1.0% in controls at 6 months and 4.9 ± 1.0 (risedronate) versus −1.0 ± 1.3 at 9 months (*p* = 0.03). No increase in hip BMD.	–
Miller and colleagues^(^ ^67^ ^)^	2011	Effects of risedronate and low‐dose transdermal testosterone on bone mineral density in women with anorexia nervosa: a randomized, placebo‐controlled study	77 women with AN	RCT	Risedronate 35 mg weekly + placebo patch versus testosterone 150 μg daily patch + weekly placebo pill versus risedronate 35 mg weekly + testosterone 150 μg daily patch versus double placebo for 12 months	12 months (study duration)	3.2% (95% CI 1.8, 4.6%; *p* < 0.0001) increase in spinal and 1.9% (95% CI 0.4, 3.4%; *p* = 0.013) BMD with risedronate compared with placebo, over 12 months. No significant effect noted with testosterone treatment	–
Resulaj and colleagues^(^ ^66^ ^)^l	2020	Transdermal estrogen in women with anorexia nervosa: an exploratory pilot study	11 premenopausal, amenorrheic women with AN	Pre‐test post‐test interventional (pilot study)	Transdermal estradiol (0.045 mg/day) and the progestin levonorgestrel (0.015 mg/day) weekly patch	6 months	Increased spinal BMD (2.0% ± 0.8%; *p* = 0.033) at 6 months. No change at the total hip or femoral neck.	–
Milos and colleagues^138^	2021	Positive effect of teriparatide on areal bone mineral density in young women with anorexia nervosa: a pilot study	10 women aged 18–35 years with AN and BMD *Z*/*T*‐score < −2.5 or fragility fracture and *Z*/*T*‐score < −1.5	Pre‐test post‐test interventional study (pilot study)	TPTD 20 μg subcutaneous for 24 months	24 months	Spinal BMD increased 13.5%, femoral neck BMD increased 5.0% and total hip 4.0%.	–
GIO								
Fujita and colleagues^139^	2000	Acute alteration in bone mineral density and biochemical markers for bone metabolism in nephrotic patients receiving high‐dose glucocorticoid and one‐cycle etidronate therapy	Patients (mean age 43.0 ± 15.7 years) with nephrotic syndrome exposed to glucocorticoids for >12 months, with spinal BMD <89% of YAM	Pre‐test post‐test interventional study	Etidronate versus no treatment	3 months	Improvement in spinal BMD with etidronate therapy (9 ± 8%, *p* = 0.003)	–
Lambrinoudaki and colleagues^(^ ^29^ ^)^	2000	Effect of calcitriol on bone mineral density in premenopausal Chinese women taking chronic steroid therapy: a randomized, double‐blind, placebo‐controlled study	81 Chinese premenopausal women with SLE, receiving glucocorticoids	RCT	0.5 μg calcitriol and 1200 mg calcium daily versus 1200 mg calcium and placebo calcitriol versus both placebo calcitriol and placebo calcium	2 years	2.1 ± 2.4% increase in spinal BMD in the intervention group compared to baseline value (*p* < 0.05), but nonsignificant when compared to calcium or placebo groups (0.4 ± 2.9% and 0.3 ± 3.5%, respectively). No significant changes were observed in any treatment group in BMD at the hip or radius.	–
Sato and colleagues^140^	2003	Effect of intermittent cyclical etidronate therapy on corticosteroid induced osteoporosis in Japanese patients with connective tissue disease: 3 year follow‐up	21–73‐year‐old adults with underlying connective tissue disease, taking >7.5 mg daily prednisolone for at least 90 days; (subgroup analysis for premenopausal women provided)	RCT	Etidronate disodium (200 mg/day for 2 weeks with 3.0 g calcium lactate and 0.75 μg alphacalcidol daily for 90 days versus 3.0 g calcium lactate and 0.75 μg alphacalcidiol daily for 90 days.	3 years	Increase in spinal BMD with etidronate versus control: +3.8 ± 6.6% versus −0.2 ± 4.8% (*p* < 0.01)	Not available for subgroups
Nakayamada and colleagues^141^	2004	Etidronate prevents high‐dose glucocorticoid induced bone loss in premenopausal individuals with systemic autoimmune diseases	16 premenopausal women and 5 men with newly diagnosed autoimmune disease and prescribed high‐dose glucocorticoids	RCT	Alfacalcidiol 1 μg/day (*n* = 11) versus alfacalcidiol and cyclical etidronate (200 mg/day for 14 days) given for 4 cycles, over 12 months	12 months	Femoral neck BMD increased 2.3 ± 1.5% in the combined group and decreased 2.5 ± 2.4% in the alfacalcidol group, *p* < 0.05	–
Nzeusseu Toukap and colleagues^142^	2005	Oral pamidronate prevents high‐dose glucocorticoid‐induced lumbar spine bone loss in premenopausal connective tissue disease (mainly lupus) patients	Premenopausal women with connective tissue disease given high‐dose glucocorticoids	RCT	Calcium (500 mg of elemental calcium/day) + vitamin D3 (25,000 units/ month) versus pamidronate (*n* = 16) 100 mg/day + calcium + vitamin D3	12 months	Reduced spinal BMD in controls (−0.045 g/cm^2^) but not in patients treated with pamidronate (−0.018 g/cm^2^) at 12 months; reduced BMD at the total hip in controls (−0.033 g/cm^2^) and in patients receiving pamidronate (−0.017 g/cm^2^)	–
Okada and colleagues^143^	2008	Alendronate protects premenopausal women from bone loss and fracture associated with high‐dose glucocorticoid therapy	47 premenopausal women commencing high dose glucocorticoid therapy for systemic autoimmune diseases	RCT	1 mg/kg/day prednisolone and alfacalcidol 1 μg/day alone (*n* = 22) versus prednisolone and alfacalcidol 1 μg/day with alendronate 5 mg/day (*n* = 25), each for 18 months.	18 months (completion of treatment)	Spinal BMD change +1.7% ± 1.4% in the combined group and −9.9% ± 1.9% in the alfacalcidol group at 12 months (*p* < 0.05). Difference also observed at 6 and 18 months.	4VF in the alfacalcidiol only group between 12–18 months
Yeap and colleagues^25^	2008	A comparison of calcium, calcitriol, and alendronate in corticosteroid‐treated premenopausal patients with systemic lupus erythematosus	Premenopausal women with SLE receiving glucocorticoids	RCT	Calcium carbonate 500 mg bd (calcium alone), calcitriol 0.25 μg bd plus calcium carbonate 500 mg bd (calcitriol + calcium), and alendronate 70 mg/week plus calcium carbonate 500 mg bd (alendronate + calcium).	2 years	Alendronate + calcium group showed significant increases in BMD of 2.69% (*p* < 0.001) in the lumbar spine and 1.41% (*p* < 0.001) in total hip. There was a 0.93% (*p* < 0.001) reduction in total hip BMD in the calcium‐alone group; there were no other significant changes.	–
Langdahl and colleagues^24^	2009	Teriparatide versus alendronate for treating glucocorticoid‐induced osteoporosis: an analysis by gender and menopausal status	Men and women with GIO (with subgroup analysis on premenopausal women)	RCT	20 μg TPTD versus alendronate 10 mg/day	18 months	Premenopausal women receiving TPTD experienced greater increments in spinal BMD (7.0% versus 0.7%, *p* < 0.001) than those prescribed alendronate.	Vertebral fractures: 0 in premenopausal women. 12 teriparatide (9 postmenopausal, 2 premenopausal, 1 man) and 8 alendronate patients (6 postmenopausal, 2 men)
Roux and colleagues^144^	2012	Post hoc analysis of a single iv infusion of zoledronic acid versus daily oral risedronate on lumbar spine bone mineral density in different subgroups with glucocorticoid‐induced osteoporosis	18–85 year old adults exposed to ≥7.5 mg/day prednisolone for <3 (prevention subgroup) or ≥ 3 months (treatment subgroup) and expected to continue glucocorticoids for >1 year; subgroup analysis provided for premenopausal women and adults aged 35–50 years	RCT	5 mg iv ZA single infusion and daily oral placebo versus 35 mg risedronate weekly and single placebo iv infusion	12 months	Greater improvement in hip BMD with ZA than risedronate in premenopausal women in both the tsreatment (*p* = 0.025) and prevention (*p* = 0.049) subpopulations. No significant differences in Risedronate versus ZA at the lumbar spine in premenopausal women. ZA significantly increased BMD at the spine at 12 months in premenopausal women, compared with risedronate, in both treatment (3.1% versus 1.7%) and prevention (1.8% versus 0.7%) subgroups.	–
IOP								
Cohen and colleagues^145^	2013	Teriparatide for idiopathic osteoporosis in premenopausal women: a pilot study	21 premenopausal women with IOP	Pre‐test post‐test interventional study (open‐label pilot study)	TPTD 20 μg daily for 18–24 months	24 months	Increase in spinal (10.8 ± 8.3% [SD]), total hip (6.2 ± 5.6%), and femoral neck (7.6 ± 3.4%) (all *p* < 0.001) BMD increased at 24 months	–
Cohen and colleagues^146^	2015	Bone density after teriparatide discontinuation in premenopausal idiopathic osteoporosis	15 premenopausal women with IOP who previously received 18–24 months of TPTD	Pre‐test post‐test	TPTD cessation	2.0 ± 0.6 years	Decline in spinal BMD 4.8 ± 4.3% (*p* = 0.0007); stable BMD at femoral neck (−1.5 ± 4.2%) and total hip (−1.1 ± 3.7%)	–
Cohen and colleagues^175^	2020	Effect of teriparatide on bone remodeling and density in premenopausal idiopathic osteoporosis: a phase II trial	41 premenopausal women with IOP	RCT	TPTD (6 months) versus placebo	24 months	6 month RCT: greater spinal BMD increase with TPTD (5.5%) versus placebo (1.5%; *p* < 0.01). 24 month follow up: TPTD increased spinal BMD (13.2%; 95% CI 10.3,16.2), total hip (5.2%; 95% CI 3.7, 6.7) and femoral neck (5%; 3.2, 6.7) at 24 months.	–
PLO								
O'Sullivan and colleagues^147^	2006	Bisphosphonates in pregnancy and lactation‐associated osteoporosis	10 women with fragility vertebral fractures presenting at a median of 1 month postpartum	Case series	Bisphosphonates (*n* = 9; 5 within 1 year of presentation)	1–19 years	Increase in spinal BMD (23%) after 2 years of treatment in women receiving bisphosphonate within 1 year of presentation (*n* = 5)	Postpartum fracture in 5 women (4/5 received bisphosphonates); *n* = 2 sustained fracture outside of pregnancy over subsequent 10 years
Choe and colleagues^125^	2012	Effect of teriparatide on pregnancy and lactation‐associated osteoporosis with multiple vertebral fractures	3 women with PLO and multiple vertebral fractures	Case series	TPTD for 18 months	18 months (completion of treatment)	Increased spinal (19.5%; range: 14.5–25%) and femoral neck (13.1%; range: 9.5–16.7%) BMD after 18 months	No fractures during period of treatment
Laroche and colleagues^148^	2017	Pregnancy‐related fractures: a retrospective study of a French cohort of 52 patients and review of the literature	52 women with fracture during pregnancy or in the 6 months post‐partum	Case control study	Bisphosphonates (*n* = 19) for 2–3 years or TPTD 20 μg (*n* = 11) for 18 months or strontium ranelate (*n* = 2) for 2 years	Mean follow‐up 2.5 years	Annual mean gain of 10.2% in spinal BMD and 2.6% at the femoral neck with bisphosphonates; annual mean gain of 14.9% in the spine with TPTD and 5.6% at the femoral neck. Annual mean gain of 6.6% at the spine and 2.3% at the femoral neck in controls	10/52 (19.2%) fractured during following (4–36 months); 3 received bisphosphonates, 1 had received TPTD. Repeat fractures during pregnancy in 2/7 who conceived again.
Hong and colleagues^124^	2018	Changes in bone mineral density and bone turnover markers during treatment with teriparatide in pregnancy‐ and lactation‐associated osteoporosis	32 women with PLO and vertebral fractures	Retrospective cohort study	TPTD 20 μg subcutaneous for 12 months (*n* = 27) versus no TPTD (*n* = 5)	12 months (to completion of treatment)	Greater increase in spinal BMD in TPTD treated women versus untreated controls (15.5 ± 6.6% versus 7.5 ± 7.1%, *p* = 0.020)	–
Lee and colleagues^149^	2021	Bone density after teriparatide discontinuation with or without antiresorptive therapy in pregnancy‐ and lactation‐associated osteoporosis	33 women with PLO	Retrospective cohort study	TPTD for a median of 12 months with (*n* = 13) or without (*n* = 20) sequential anti‐resorptive therapy ((alendronate [*n* = 3], denosumab [*n* = 4], ibandronate [*n* = 3], risedronate [*n* = 2], and zoledronic acid [*n* = 1]; median 18 months)	Median 18 months post course of TPTD	No difference in mean spinal BMD between patients treated with antiresorptive therapy versus those not treated with antiresorptives.	No fractures in subsequent pregnancies
Lampropoulou‐Adamidou and colleagues^150^	2021	Teriparatide treatment in patients with pregnancy‐and lactation‐associated osteoporosis	19 premenopausal women with PLO	Retrospective cohort study	TPTD + calcium + vitamin D (*n* = 19) versus calcium + vitamin D (*n* = 8) for 24 months	24 months (to completion of treatment)	aBMD increase of 20.9 ± 11.9% (TPTD) versus 6.2 ± 4.8% (control) at the LS (*p* < 0.001), 10.0 ± 11.6% versus 5.8 ± 2.8% at the TH (*p* = 0.43)	Median of 4.0 (3–9) VFs in TPTD group versus 2.5VFs (1–10) *p* = 0.02
Solid‐organ tumors								
Gnant and colleagues^151^	2007	Zoledronic acid prevents cancer treatment‐induced bone loss in premenopausal women receiving adjuvant endocrine therapy for hormone‐responsive breast cancer: a report from the Austrian Breast and Colorectal Cancer Study Group	401 premenopausal women with hormone‐responsive breast cancer	RCT	Tamoxifen (20 mg/d orally) and goserelin (3.6 mg every 28 days subcutaneously) ± ZA (4 mg iv 6 monthly) versus anastrozole (1 mg/d orally) and goserelin ± ZA for 3 years	3 years	ZA associated with stable BMD while patients not given ZA demonstrated bone loss after 3 years (−14.4%, *p* < 0.0001)	–
Gnant and colleagues^41^	2008	Adjuvant endocrine therapy plus zoledronic acid in premenopausal women with early‐stage breast cancer: 5‐year follow‐up of the ABCSG‐12 bone‐mineral density substudy	Premenopausal women with endocrine‐responsive breast cancer receiving adjuvant endocrine therapy (goserelin and anastrozole or goserelin and tamoxifen)	RCT	4 mg intravenous ZA every 6 months, then 4 mg every 6 months over 3 years (*n* = 899) versus no treatment (*n* = 904)	60 months (median; range 15.5–96.6)	Greater BMD loss with anastrazole than tamoxifen, in patients not receiving ZA. (spinal −13.6% versus −9.0%, *p* < 0.0001). 2 years post treatment with ZA, decreased BMD again noted in those not exposed to ZA (spinal BMD ‐6.3%) while patients who received ZA had stable BMD at 36 months (+0.4%, trochanter at spine, +0.8% at trochanter) and increased BMD at 60 months (+4.0% at spine and + 3.9% at trochanter)	–
Hershman and colleagues^152^	2008	Zoledronic acid prevents bone loss in premenopausal women undergoing adjuvant chemotherapy for early‐stage breast cancer	101 premenopausal women with breast cancer undergoing adjuvant chemotherapy	RCT	ZA 4 mg iv 3 monthly versus placebo for a year	1 year	In patients randomized to ZA, BMD was stable at the LS (−0.03% at 24 weeks, −0.6% at 52 weeks), FN (+0.2% at 24 weeks, +0.4% at 52 weeks), and TH (−0.19 at 24 weeks, −0.12% at 52 weeks). In contrast, BMD was reduced with at the spine (−2.98% at 24 weeks and −4.39%) and total hip (−2.08% at 52 weeks) with placebo, significantly (*p* < 0.05) different to ZA.	–
Hines and colleagues^153^	2009	Phase III randomized, placebo‐controlled, double‐blind trial of risedronate for the prevention of bone loss in premenopausal women undergoing chemotherapy for primary breast cancer.	216 premenopausal women undergoing adjuvant chemotherapy for breast cancer	RCT	Calcium 600 mg, Vitamin D 400 IU + risedronate 35 mg weekly or placebo	12 months	No difference in mean BMD change at the spine (4.3% risedronate, 5.4% placebo at 1 year; *p* = 0.18) or femoral neck.	–
Kim et al	2010	Zoledronic acid prevents bone loss in premenopausal women with early breast cancer undergoing adjuvant chemotherapy: a phase III trial of the Korean Cancer Study Group (KCSGBR06‐01)	112 premenopausal women aged >40 years undergoing adjuvant chemotherapy for early stage hormone + breast cancer	RCT	4 mg iv ZA 6 monthly (*n* = 56) versus observation (delayed ZA till fragility fracture or spinal/hip BMD *T*‐score ≤ −2.5 at 6 month review (*n* = 56))	12 months (treatment completion)	Stability in spinal (+0.5% ± 3.2% at 6 months, −1.1 ± 3.7% at 12 months) and femoral neck (+0.4 ± 4.4% at 6 months and + 1.1 ± 5.6% at 12 months) BMD with ZA and reduction in BMD at spinal (−3.1 ± 4.8%, *p* < 0.001 at 6 months and −7.5 ± 2.8%, *p* < 0.001 at 12 months) and femoral neck (−1.1 ± 3.4%, *p* = 0.044 at 6 months and −3.4 ± 3.3%, *p* < 0.001 at 12 months) BMD with observation	–
Kim and colleagues^154^	2011	Zoledronic acid prevents bone loss in premenopausal women with early breast cancer undergoing adjuvant chemotherapy: a phase III trial of the Korean Cancer Study Group (KCSG‐BR06‐01)	Premenopausal >40 years women with early breast cancer receiving adjuvant chemotherapy	RCT	Upfront ZA 4 mg iv 6 monthly (*n* = 57) versus delayed ZA (observation; *n* = 59)	12 months	ZA prevented spinal BMD loss (−1.1% with ZA versus −7.5% with observation group at 12 months. Between group difference of difference in % change from baseline: Differences 6.4% for the LS, and 3.6% for the femoral neck.	–
Shapiro and colleagues^42^	2011	Zoledronic acid preserves bone mineral density in premenopausal women who develop ovarian failure due to adjuvant chemotherapy: final results from CALGB trial 79809	439 premenopausal women with chemotherapy induced ovarian failure	RCT	ZA 4 mg 3 monthly for 2 years commenced within 1–3 months or after 1 year of chemotherapy initiation	3 years	Less bone loss at 12 months in women randomized to ZA at initiation of chemotherapy (median + 1.2% versus −6.7%, *p* < 0.001 in women with chemotherapy induced ovarian failure, and median + 1.4% versus −5.5, *p* < 0.001 in all women). Less bone loss at 3 years in women who received ZA at initiation of chemotherapy (median + 1% versus ‐0.5%, *p* = 0.019).	–
Hadji and colleagues^155^	2014	Effects of zoledronic acid on bone mineral density in premenopausal women receiving neoadjuvant or adjuvant therapies for HR+ breast cancer: the ProBONE II study	70 premenopausal women with early BC receiving adjuvant chemotherapy and/or endocrine therapy	RCT	4 mg iv ZA (*n* = 34) versus placebo (*n* = 36) over 2 years	24 months (treatment completion)	Increased spinal BMD 3.14% from baseline to 24 months with ZA versus a 6.43% decrease with placebo *p* < 0.0001). Increased BMD also at the femoral neck (right:1.22% and left: 0.74%) while reduction in BMD seen at these sites (−2.38% *p* < 0.0001 and −2.28%, *p* < 0.002).	1 traumatic rib fracture (ZA)
Kalder and colleagues^156^	2015	Effects of zoledronic acid versus placebo on bone mineral density and bone texture analysis assessed by the trabecular bone score in premenopausal women with breast cancer treatment‐induced bone loss: results of the ProBONE II substudy	70 premenopausal women with ER positive and/or PR positive BC considered for adjuvant/neoadjuvant chemotherapy and/or adjuvant endocrine therapy	RCT	4 mg iv ZA 3 monthly (*n* = 34) versus placebo (*n* = 36) for 2 years	24 months (treatment completion)	Significant increase in spinal BMD with ZOL at 12 and 24 months (2.17%, *p* < 0.001 and 3.14%, *p* < 0.001). Significant decrease in spinal BMD with placebo at 12 and 24 months (−5.02%, *p* < 0.001 and 6.43%, *p* < 0.001).	–
Kyvernitakis and colleagues^157^	2018	Prevention of breast cancer treatment‐induced bone loss in premenopausal women treated with zoledronic acid: final 5‐year results from the randomized, double‐blind, placebo‐controlled ProBONE II trial.	Premenopausal women with early BC receiving adjuvant chemotherapy and/or endocrine therapy	RCT	4 mg iv ZA every 3 months (*n* = 34) versus placebo (*n* = 36) over 2 years	60 months	ZA prevented treatment‐induced bone loss: Spinal BMD increase by 2.9% with ZA versus 7.1% decrease in placebo‐treated patients. Over 60 months, 2.2% decrease in spinal BMD in ZA patients versus 7.3% decline in placebo‐treated patients (*p* < 0.001)	–
Coleman and colleagues^158^	2021	Bone health outcomes from the international, multicenter, randomized, phase 3, placebo‐controlled D‐CARE study assessing adjuvant denosumab in early breast cancer	Women with stage II/III breast cancer receiving neo/adjuvant chemotherapy. Subgroup analysis based on menopausal status provided.	RCT	Denosumab (*n* = 2256) or placebo (*n* = 2253) 3–4 weekly for 6 months and then every 3 months for 5 years	5 years from commencement	‐	HR for time to on‐study fracture 0.74 (0.56–0.99) in favor of denosumab
Other								
Palomba and colleagues^159^	2002	Raloxifene administration in women treated with gonadotropin‐releasing hormone agonist for uterine leiomyomas: effects of bone metabolism	100 premenopausal women with uterine leiomyomas treated with leuprolide acetate	RCT	Raloxifene 60 mg/day versus placebo	42 weeks	No change in BMD at 42 weeks in women treated with raloxifene; ~1%/month reduction in BMD noted in women not treated with raloxifene	–
Mitwally and colleagues^160^	2002	Prevention of bone loss and hypoestrogenic symptoms by estrogen and interrupted progestogen add‐back in long‐term GnRH‐agonist down‐regulated patients with endometriosis and premenstrual syndrome	15 premenopausal women with endometriosis and 5 with severe premenstrual syndrome (PMS) receiving leuprolide depot 1.75 mg im	Pre‐test post‐test study	1 mg oral micronized estradiol daily and 0.35 mg norethindrone daily for 2 days alternating with 2 days without norethindrone.(commenced at 2–3 months for endometriosis patients and 1 month for PMS patients)	31.2 ± 17 months (for endometriosis patients) and 37.7 ± 8.4 (PMS patients)	No significant change in spinal or femoral neck BMD	–
Cundy and colleagues^161^	2003	A randomized controlled trial of estrogen replacement therapy in long‐term users of depot medroxyprogesterone acetate	38 premenopausal women with min. 2 year DMPA use and spinal BMD *T*‐score ≤ 0	RCT	0.625 mg conjugated estrogen versus placebo	24 months	Increase in spinal BMD in the estrogen‐treated group (1%) and reduction in BMD in the placebo group (−2.6%; 3.5% between‐group difference at 24 months). No significant difference at the femoral neck.	–
Aris and colleagues^162^	2004	Efficacy of alendronate in adults with cystic fibrosis with low bone density	Adults with CF	RCT	Alendronate 10 mg/day (*n* = 24) orally versus placebo (*n* = 24) for 1 year	12 months	Increased spinal (4.9 ± 3.0%; *p* < 0.001) and hip (2.8 ± 3.2%, *p* = 0.003) BMD with alendronate. Loss of BMD at the spine (−1.8 ± 4.0%) and hip (−0.7 ± 4.7%).	–
Ripps and colleagues^163^	2003	Alendronate for the prevention of bone mineral loss during gonadotropin‐releasing hormone agonist therapy	11 premenopausal women commenced on GnRHa therapy	RCT	Alendronate 10 mg/d versus placebo for 6 months	6 months	Mean increase in spinal BMD of 1.0% (*p* = 0.35) with alendronate and a loss of 3.8% (*p* = 0.01) with placebo. Stable hip BMD with alendronate (−0.4%, *p* = 0.65) versus reduction in BMD (−3.4%, *p* = 0.02) with placebo..	–
Adami and colleagues^164^	2009	Intravenous neridronate in adults with osteogenesis imperfecta	23 men and 23 premenopausal women (18–50 years) with OI	RCT	100 mg iv neridronate 3 monthly versus no treatment (calcium and vitamin D supplemented if deficient)	24 months	Increase in spinal (3.0 ± 4.6% (SD)) and hip BMD (4.3 ± 3.9%), in neridronate treated patients within the first 12 months versus no significant change in untreated patients,	VF RR in neridronate treated patients 0.14 (*p* = 0.03)
Chapman and colleagues^165^	2009	Intravenous zoledronate improves bone density in adults with cystic fibrosis	22 adults with CF (non‐transplanted)	RCT	2 mg iv ZA (*n* = 10) or placebo (*n* = 12) 3 monthly for 2 years. All received calcium and vitamin D	2 years from commencement	Spinal BMD increase greater with ZA (6.14% ± 1.86) than placebo (0.44 ± 0.10; *p* = 0.021) at 24 months. Similar findings at the femoral neck (4.23% ± 1.3 versus −2.5% ± 1.41, *p* = 0.0028)	Nil fractures in either group
Kacker and colleagues^166^	2014	Bone mineral density and response to treatment in men younger than 50 years with testosterone deficiency and sexual dysfunction or infertility	75 men aged <50 years with total testosterone <12.1 nmol/L or free testosterone with sexual dysfunction or infertility	Cohort study	Testosterone cypionate (*n* = 43) at initial dose of 100 mg weekly or 200 mg biweekly OR testosterone pellets 750 – 900 mg 3 monthly versus clomiphene citrate (*n* = 17) 25 mg daily or 50 mg 3 times a week versus anastrazole (*n* = 3)	30.4 ± 16.2 months	Increased spinal (+0.0306 ± 0.0392 g/cm^2^, *p* < 0.001) but not hip BMD in men treated with testosterone; reduced spinal BMD) in men treated with clomiphene citrate (−0.0144 ± 0.0199, *p* = 0.0089). No significant change (−0.058 ± 0.0331, *p* = 0.094 spinal BMD; 0.0145 ± 0.0462, *p* = 0.76 total hip BMD) in men treated with anastrazole	–
Yassin. and colleagues^167^	2014	Effects of the anti‐receptor activator of nuclear factor kappa B ligand denosumab on beta thalassemia major‐induced osteoporosis	30 patients aged 18–32 years with beta‐thalassemia major induced osteoporosis	Pre‐test post‐test interventional study	60 mg subcutaneous denosumab 6 monthly	12 months	9.2% (95% CI 8.2–10.1%) increase in spinal BMD at 12 months and 6.0% (95% CI 5.2–6.7%) increase at the femoral neck	–

AN = anorexia nervosa; BC = breast cancer; bd = twice per day; BMD = bone mineral density; CF = cystic fibrosis; CI = confidence interval; DMPA = depot medroxyprogesterone; ER = estrogen receptor; GnRHa = gonadotropin releasing hormone agonist; IOP = idiopathic osteoporosis; iv = intravenous; OI = osteogenesis imperfecta; PLO = pregnancy and lactation associated osteoporosis; PMS = premenstrual syndrome; PR = progesterone receptor; RCT= randomized controlled trial; SD = standard deviation; SLE = systemic lupus erythematosus; TPTD = teriparatide; VF = vertebral fracture; YAM = young adult mean; ZA= zoledronic acid.

#### Safety of available therapy in premenopausal women and men

Anti‐osteoporosis therapies are not approved for use in pregnant or lactating women. Use of bisphosphonates is cautioned in premenopausal women due to a theoretical teratogenic effect, because bisphosphonates cross the placenta. Animal studies have demonstrated potential adverse impact on fetal long‐bone growth with exposure to bisphosphonates.^(^ ^168^ ^)^

A review of 36 women who were exposed to bisphosphonates duriSecondary osteoporosis ng or in the 6 weeks prior to pregnancy revealed no increase in congenital abnormalities in the offspring, compared with offspring of healthy women and women with systemic disease unexposed to bisphosphonates.^(^ ^169^ ^)^ This is consistent with another review that evaluated 51 women exposed to bisphosphonates prior to or during pregnancy; no teratogenic effects were identified.^(^ ^170^ ^)^ A case‐series reported 20% rate of congenital abnormalities; however, no control group was available.^(^ ^171^ ^)^

Teriparatide has even more limited safety data in premenopausal women, although lack of skeletal retention is reassuring. Ethical considerations clearly limit the plausibility of RCTs to assess the safety of these therapies in women planning for pregnancy. The lack of safety data does, however, complicate the management of osteoporosis in YAs.

### Therapeutics for IOP

Weight‐bearing exercise has recognized benefits in assisting in the management of postmenopausal osteoporosis. In a small cohort of premenopausal women with IOP, 30 minutes of aerobic exercise three times a week and one to two sessions of strength and aerobic training per week resulted in increased spinal aBMD.^(^ ^172^ ^)^

The benefits of alendronate, parathyroid (PTH) analogues, and denosumab have been evaluated in IOP. In middle‐aged men with IOP in the setting of low BMD or fragility fractures, alendronate in addition to calcium and vitamin D increased spinal aBMD (2.7%/year).^(^ ^173^ ^)^ Increases at the total hip were also noted, but not at the femoral neck.^(^ ^173^ ^)^ Treatment with PTH analogues in a small group of men (aged 30–68 years) with IOP led to improvements in both the femoral neck (2.9%) and spinal aBMD.^(^ ^174^ ^)^ Studies focusing on younger men and fracture risk, are limited.

In premenopausal women with IOP, teriparatide increased aBMD by 5.5% and significantly more than placebo at 6 months in a phase II RCT.^(^ ^175^ ^)^ Teriparatide also increased cancellous and endocortical bone forming rates 3.3‐fold and fourfold respectively, at 3 months.^(^ ^175^ ^)^ Increases in aBMD and TBS persisted at 24 months, with average spine BMD increases of 13.2%.^(^ ^175^ ^)^ Phase II studies for denosumab therapy post–teriparatide treatment are under way. Safety of denosumab and the appropriate consolidation therapy to prevent rebound vertebral fractures following denosumab cessation in YAs also requires due consideration.

### Suggested approach to osteoporosis in YAs

**Fig. 2:**
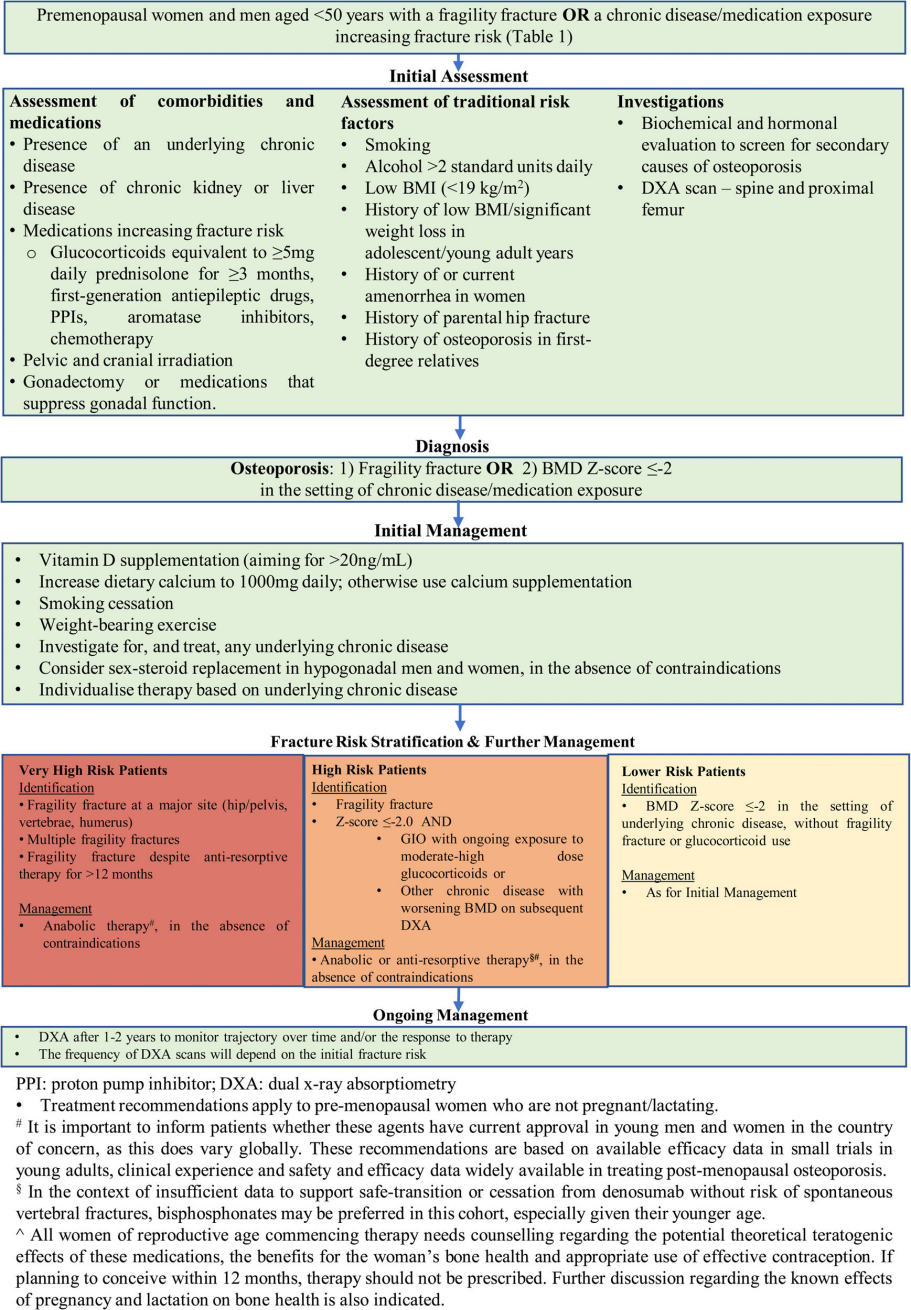
Osteoporosis management in premenopausal women and men aged <50 years.

In the absence of a substantial body of research, the following recommendations are based on currently available evidence and clinical experience. Figure 2 provides an algorithm for the appropriate assessment and management of osteoporosis in YAs.

As noted in Fig. 2, we recommend that all YAs with fragility fracture (excluding fractures of the skull and digits) undergo clinical and biochemical (Table 3) assessment for causes of secondary osteoporosis, and assessment with DXA. Where possible, use of local normative data for YAs, referenced for sex, is recommended to negate the impact of variation due to sex and ethnicity. As the significance of idiopathic low BMD is yet to be elucidated, we recommend against screening YAs with DXA in the absence of fragility fracture or established risk factors for fracture.

**Table 3:** Recommended Biochemical Screening for Secondary Osteoporosis in Younger Adults

**Parameters related to bone mineralization and accrual** Vitamin D level, PTH, Corrected calcium, magnesium, phosphate
**Screening for secondary causes** LH, FSH, prolactin, estradiol or testosterone Urea, electrolytes and creatinine Liver function tests Serum protein electrophoresis Serum tryptase Anti‐tissue transglutaminase antibodies TSH, T4 HbA1c ESR Bone specific ALP
**Selective screening, based on clinical history and examination** 24‐Hour urinary cortisol (if Cushing's syndrome suspected) 24‐Hour urinary calcium (in those with a history of renal stones) Growth hormone, IGF‐1 levels Hepatitis and HIV serology

ALP = alkaline phosphatase; ESR = erythrocyte sedimentation rate; FSH = follicle‐stimulating hormone; HbA1c = glycated hemoglobin A1c; HIV = human immunodeficiency virus; IGF‐1 = insulin like growth factor‐1; LH = luteinizing hormone; PTH = parathyroid hormone; T4 = free thyroxine; TSH = thyroid stimulating hormone.

We suggest a diagnosis of osteoporosis in YAs if either of the following criteria are met:

1.  Fragility fracture in the setting of chronic disease/medication exposure (Table 1)
2.  BMD of *Z*‐score <2 or *T*‐score < −2.5 at the lumbar spine or hip in the setting of chronic disease/medication exposure (Table 1) regardless of fracture status.

The role of BMD in the diagnosis of osteoporosis in YA with fractures in the absence of clinical risk factors is unclear. Given previously described evidence to support the presence of microarchitectural abnormalities in YAs with fragility fracture and varying aBMD (with average aBMD *Z*‐scores > −2.0 at the spine and hip) in the absence of clinical risk factors, consideration of fragility fracture alone as a diagnostic criterion for osteoporosis in YAs is relevant. Further research to investigate the implications of unexplained fragility fracture without low BMD in YAs is required.

A distinction between sites of fragility fracture have not been made, other than to exclude the skull and digits, because premenopausal fracture at various sites, including ankle, wrist, and leg, have been associated with increased risk of postmenopausal fracture.^(^ ^176^ ^)^ However, we acknowledge that some fractures, such as at the hip, spine, radius, or humerus, are more clinically significant.

In YAs with secondary osteoporosis, optimization of existing risk factors for bone loss is recommended in the first instance (see Fig. 2). This may include optimization of vitamin D levels and dietary or supplemental calcium intake, commencement of weight‐bearing exercise and correction of hypogonadism if safe to do so, correction of factors such as hypercalciuria if relevant, reducing exposure to offending medications, and optimization of the underlying disease.

In patients with high risk of fracture, however, such as those who have sustained fractures at major sites (such as the hip or vertebrae), multiple osteoporotic fractures, or osteoporotic fractures in the presence of chronic disease, or those who have significant loss of bone (>5% or 0.045 g/cm^2^ per year of aBMD at the lumbar spine or total hip/femoral neck), consideration of anti‐osteoporosis therapy would be prudent. Anabolic therapy may be especially suitable for those patients with very high risk of fracture (Fig. 2) and those with multiple fractures or fracture at major sites such as the vertebrae or hip. However, prescription is likely to be affected by regional factors such as cost and availability. Alternatively, anti‐resorptive therapy can be considered.

In women of reproductive age, discussion of family planning is of the utmost importance prior to commencing anti‐osteoporotic therapy. In considering anti‐resorptive therapy, oral bisphosphonates are preferred over intravenous therapy or denosumab, given the shorter half‐life of risedronate and the absence of rebound fracture risk following withdrawal. Patient education of the potential adverse effects anti‐resorptives on the fetus and offspring skeleton is needed. Anti‐resorptive therapy should not be commenced in women planning to conceive within the next 12 months. Clinical experience suggests that oral bisphosphonate therapies, provided they are ceased at least 12 months prior to conception, are not associated with increased risk of teratogenicity.

### Implications for further research

Further research into the incidence, risk stratification, and management of YAs is clearly needed. The implications of and the pathophysiology underlying idiopathic fracture in YAs need to be characterized. The significance of isolated low BMD in YAs is as yet unclear. Certainly, available data suggest that there is abnormal bone microarchitecture in these individuals. However, their long‐term fracture risk and thus an intervention threshold, has not been established.

Targeted tools for fracture risk calculation and guidance on the optimal methods of diagnosing and monitoring osteoporosis in YAs are needed. Fracture risk assessment tools should ideally take into consideration the presence of different chronic diseases, sex, and ethnicity.

Research on the safety and efficacy of therapeutic agents in this cohort is currently limited. Furthermore, the benefits and disadvantages of the current common practice of preferential use of anti‐resorptives over anabolic therapy in YAs requires elucidation.

Dedicated research into the above areas of clinical need will assist in the development of guidelines, which would benefit both YA patients and their healthcare professionals.

## Conclusion

Fragility fractures in YAs are uncommon. However, an increasing body of research demonstrates pathological bone microarchitecture and/or low aBMD with or without fracture in a subpopulation of young men and women. Although the implications of idiopathic low BMD on long‐term fragility fracture risk are less well‐established, fractures in those with IOP and in those with or without chronic disease warrant expert management. A stronger research focus into optimizing the diagnosis, monitoring, and management of osteoporosis in YAs is now needed.

## Conflict of Interests

MH has no conflicts of interest or disclosures to declare. AC has received research support from Eli Lilly and Amgen. PRE has received research funding form Amgen, Eli‐Lilly, and Alexion, and honoraria from Amgen. FM has no conflicts of interest or disclosures to declare.

### Peer Review

The peer review history for this article is available at https://publons.com/publon/10.1002/jbm4.10594.

## Supporting information


**Appendix S1**. Supporting information.
