# Supplementary material for: Dilemmas in the Management of Osteoporosis in Younger Adults
Source: JBMR Plus. 2022 Jan 19;6(1):e10594. doi: 10.1002/jbm4.10594 (PMC8771004; doi:10.1002/jbm4.10594)
Supplement: Supplementary file 1 — Appendix S1. Supporting information. [file JBM4-6-e10594-s001.doc]

**SUPPORTING INFORMATION
LITERATURE SEARCH METHOD**

Review of the literature available from January 2000 – August 2021 was systematically searched using the same search strategy on PubMed, Embase and Web of Science.

Title/Abstract search for:

1. Idiopathic osteoporosis AND diagnosis
2. Osteoporosis AND cause* AND young
3. Osteoporosis AND pre-menopause*
4. Minimal trauma fracture AND young
5. Bisphosphonate AND young adult AND osteoporosis
6. Teriparatide AND osteoporosis AND young adult
7. Denosumab AND young adult AND osteoporosis
8. Osteoporosis AND pregnancy
9. Breastfeed* AND bisphosphonate
10. Breastfeed AND teriparatide
11. Breastfeed AND denosumab
12. Osteoporosis AND diagnos* AND young adult
13. Fracture risk assessment AND young
14. DXA AND fracture risk AND young
15. HRpQCT AND young
16. Hip Structural Analysis AND young
17. Trabecular bone score AND young
18. Osteoporosis AND estrogen
19. Osteoporosis AND testosterone
20. Osteoporosis AND young AND guide*
21. Osteoporosis AND young AND statement
22. Osteoporosis AND young AND white paper
23. Premenopaus* AND Teriparatide OR denosumab OR zoledron* OR risedronate OR alendronate* OR etidronate
24. Breast cancer AND zoledron* OR alendronate OR denosumab* OR teriparatide OR risedronate OR etidronate
25. Lupus AND zoledron* OR alendronate OR denosumab OR teriparatide OR risedronate OR etidronate
26. Inflammatory bowel disease AND zoledron* OR alendronate OR denosumab OR teriparatide OR risedronate OR etidronate
27. Crohn* AND zoledron* OR alendronate OR denosumab OR teriparatide OR risedronate OR etidronate
28. Ulcerative colitis AND zoledron* OR alendronate OR denosumab OR teriparatide OR risedronate OR etidronate
29. Glucocorticoid* AND zoledron* OR denosumab OR alendronate OR teriparatide OR risedronate OR etidronate OR romosozumab

Inclusion criteria

- Timeline 2000 January to 2021 August
- Case series, case–control studies, cohort studies, non-randomized intervention trials and RCTs as well as systematic reviews with or without meta-analyses, position statements and guidelines and prior literature/narrative reviews (expert opinion)
- Demonstrates a clear relationship between exposure and fracture/bone density/HRpQCt data
- Addresses osteoporosis/fracture risk in premenopausal women/younger adults, specifically

Exclusion criteria

- Case reports, conference abstracts
- Articles which consider benefits and disadvantages of surgical fixation of fractures
- Articles which focus on stress fracture
- Focus on perimenopausal women
- Articles focusing on osteomalacia
- Studies which included pre-menopausal women only as baseline data for assessment of postmenopausal women
- Full text unavailable
- Animal studies focusing on interventions
- Studies which look at variability in markers in healthy adults
